# Cellular senescence—from solid organs to vascularized composite allotransplants

**DOI:** 10.1007/s11357-025-01788-2

**Published:** 2025-07-28

**Authors:** Leonard Knoedler, Andreas Schroeter, Jasper Iske, Jillian Dean, Sam Boroumand, Thomas Schaschinger, Tobias Niederegger, Samuel Knoedler, Adriana C. Panayi, Max Heiland, Stefan G. Tullius, Bohdan Pomahac, Martin Kauke-Navarro

**Affiliations:** 1 Department of Oral and Maxillofacial Surgery, Charité–Universitätsmedizin Berlin, Corporate Member of Freie Universität Berlin and Humboldt-Universität zu Berli, Augustenburger Platz 1, 13353 Berlin, Germany; 2 Division of Plastic Surgery, Department of Surgery, Yale New Haven Hospital, Yale School of Medicine, New Haven, CT USA; 3 Hannover Medical School, Department of Plastic, Aesthetic, Hand and Reconstructive Surgery, Burn Center, Hannover, Germany; 4 Division of Transplant Surgery, Department of Surgery, Brigham and Women’s Hospital, Harvard Medical School, Boston, MA USA; 5 Department of Cardiothoracic and Vascular Surgery, Deutsches Herzzentrum Der Charité, Berlin, Germany; 6 Charité Universitätsmedizin Berlin, Berlin, Germany; 7 School of Medicine, University of Pittsburgh, Pittsburgh, PA USA

**Keywords:** Vascularized composite allotransplantation, VCA, Transplant, Reconstructive surgery, Senescence, Senotherapeutics, Immune aging, Immunosenescence

## Abstract

Vascularized composite allotransplantation (VCA) has emerged as a novel therapy approach to restore form and function in patients with severe tissue defects of the face, hand, and abdominal wall, among other anatomical regions. The composite allografts comprise different tissues such as skin, muscle, or bone. Clinical data demonstrate promising mid- and long-term outcomes following VCA surgery, but our understanding of the cellular interactions and molecular pathways in VCA surgery is oftentimes deduced from solid organ transplantation (SOT). In SOT, the concept of cellular senescence has grown increasingly popular which is characterized by a permanent cellular proliferation arrest in response to endogenous and exogenous stimuli. Senescent cells, through the release of mitochondrial DNA and secretion of proinflammatory proteins, can amplify the immunogenicity of transplants, hindering graft acceptance and longevity. This understanding has paved the way for novel interventions, including the use of senolytics—agents that selectively eliminate senescent cells—to modulate immune responses and mediate immunotolerance. There is a body of evidence that underlines the therapeutic potential of senescence to improve SOT outcomes; however, the relevance of senescence to VCA outcomes remains elusive. In this review, we aim to summarize the current literature on senescence in different solid organ transplants and outline the potential impact of senescence on VCA outcomes. This knowledge may help providers develop a broader understanding of the cellular and molecular landscape in VCA to develop targeted therapies and advance VCA patient care.

## Introduction

Vascularized composite allotransplantation (VCA) represents an emerging era in transplant surgery to restore form and function with composite allografts [1–3]. Expanding the boundaries of conventional reconstructive approaches, VCA surgery has become a viable therapeutic option for patients with devastating injuries or irreversible tissue loss such as severe midfacial disfigurement or extremity amputations [4, 5]. Since its advent in the 1990 s, a growing number of VCA procedures including full-face and hand transplantations have been performed, with promising mid- and long-term outcomes [6, 7]. The transplanted units comprise a heterogeneous set of different tissues ranging from skin and muscle through bone to vasculature and nerves. Notably, this unique composition, with presumed component-specific antigenicity, carries the risk of strong immune responses [8–10].

While previous research work had focused on skin tissue immunogenicity, the oral and nasal mucosa have recently been identified as another antigenic structure of VCA grafts^11^. Due to the immunogenicity of the different tissue types transplanted en bloc, VCA patients require lifelong immunosuppression to prevent graft rejection and ensure durable surgical success [11]. The underlying mechanisms of VCA rejection are subject to ongoing research. Understanding the cellular network in VCA allografts provides a possible lever for modulating the immunogenic potential of VCA transplants [12]. For example, regulatory T cells have been demonstrated to attenuate the alloreactive immune response and induce immunotolerance [3]. While distinct cell types such as T cells, epidermal and follicular stem cells, and endothelial cells appear as the main protagonists in VCA skin rejection episodes, the specific cell fate and state remain poorly understood [12].

In the field of solid organ transplantation (SOT), the concept of senescence has gained scientific interest over the past decades [13]. Cellular senescence describes a permanent cellular proliferation arrest in response to endogenous and exogenous stresses and harms (e.g., oncogene activation, telomere shortening, or DNA damage) [14, 15]. In principle, cellular senescence is a physiological process and crucial for suppressing the unregulated growth of degenerated cells [16]. Between 30 and 70% of senescent cell populations have been shown to secrete pro-inflammatory, pro-apoptotic, and pro-fibrotic factors, termed the senescence-associated secretory phenotype (SASP), while the other portion can secrete growth and regenerative factors such as VEGF-A and PDGF-AA [17]. Yet, senescent cells can release mitochondrial DNA and secrete proinflammatory proteins potentiating the immunogenicity of solid organ transplants [18]. Emerging insights into the senescence cascade revealed that senolytics (i.e., drugs that selectively clear senescent cells) could dampen posttransplantation immune responses and prolong transplant survival [19].

Building on advances in solid organ transplantation (SOT), we hypothesize that targeting cellular senescence may address unique immunologic challenges in VCA. Although research in senescence modulation has shown potential to reduce immune rejection and prolong graft survival in SOTs [20, 21], its implications for VCA remain largely uncharted. Given the distinctive tissue composition and immunogenicity of VCA grafts, investigating senescence as a modifiable factor could yield new therapeutic approaches to enhance transplant durability and patient outcomes. Herein, we aim to summarize the current body of evidence on senescence in SOT and deduce potential implications for VCA transplants. A comprehensive overview and better understanding of cellular senescence in transplant surgery may facilitate the development of targeted therapeutics and overcome persisting hurdles in VCA surgery.

## Senescence in solid organ transplantation

### The molecular signature of cellular senescence

Cellular senescence is defined by a complex molecular signature, including a pool of cellular markers. From this pool, different cellular markers have been used to define and demask cellular senescence, depending on histological specificity, preservation modality, and the dichotomy of in vitro versus in vivo analytical frameworks [22]. Senescent cells have been demonstrated to display an overexpression of endogenous lysosomal beta-galactosidase, termed senescence-associated beta-galactosidase (SA**-**βgal) [23]. For example, Xu et al. transplanted 1 × 10^6^ radiation-induced senescent (SEN) pre-adipocytes into 6-month-old mice by intraperitoneal injection. This transplantation led to a marked decrease in physical functionality—such as reduced maximal walking speed, hanging endurance, and grip strength—within just one month post-transplantation, compared to controls receiving non-senescent (CON) cells. The study further showed that even smaller doses of SEN cells in older mice similarly induced physical decline and were associated with reduced survival. Interestingly, the functional decline correlated with increased systemic levels of SA-βgal^+^ and p16I^nk4a^ mRNA levels. p16^INK4A^ is a cyclin-dependent kinase inhibitor that serves as a cell cycle regulator. Moreover, the introduction of senolytic drugs, dasatinib plus quercetin, effectively cleared senescent cells, reduced proinflammatory cytokine secretion in human adipose tissue explants, and improved physical function and survival in both senescent cell-transplanted and naturally aged mice [24]. Paralleling with other regulators such as p21^Cip1^ and p53, the accumulation of p16^INK4A+^ cells has been correlated with senescence [25]. More precisely, p16^INK4A^ and p21^Cip1^ have been shown to be upregulated in skin and cardiac tissue of aging mice. In a supporting manner, transplanting murine senescent pre-adipocytes has been found to increase the number of p16^INK4A+^ cells in the recipient mice [18]7/19/2025 8:01:00 AM. The independent upregulation of p16 and p21Cip1 has been investigated through the generation of a human dermal fibroblast (HDF) cell strain with a fluorescent reporter for p21. This research revealed that as cells approach senescence, the expression of p16 and p21, alongside SA-βgal, increases, with a corresponding decrease in DNA synthesis as indicated by BrdU incorporation [26]. Notably, Herbig et al. demonstrated that, at 10 remaining population doublings (RPD), 79% of cells were actively cycling and 80% were negative for both p16^INK4A^ and p21^Cip1^ expression. As the cells progressed closer to senescence (reaching two RPDs), the fraction of non-cycling cells rose from 21 to 63%, yet 70% of these cells remained positive for only one of p16^INK4A^ or p21^Cip1^, highlighting the need for moving beyond isolated towards holistic analysis of cellular senescence [27]. Further research disclosed that, while approximately 10% of early passage cells exhibited γ-H2AX foci, this percentage increased to 90% in senescent cells. Conversely, the immortalization of cells with telomerase (hTERT) led to a marked decrease in γ-H2AX-positive cells, dropping to 7% [28]. Therefore, γ-H2AX foci can also help determine cellular senescence. In addition, various cytokines such as IL‐1α, IL‐1β, IL-6, IL-8, and TNF-α can define senescent cell states [29]. For example, experimental murine models showed that transplantation of senescent renal scattered tubular-like cells not only led to increased creatinine levels and renal tissue hypoxia but also induced IL-6 gene expression [30]. Besides cytokines, CXCR2, IGF2, chemokines, and metalloproteases have been used to determine cellular senescence [31].

### Molecular, genomic, and cellular senescence drivers

Since the seminal findings by Hayflick and Moorfield in 1961, research has identified different drivers of senescence, including molecular, genomic, and cellular triggers [32]. For example, DNA damage has been proposed as a leading cause of cellular senescence [33]. More precisely, in actively dividing cells, the progressive shortening of telomeres has been demonstrated to culminate in the exposure of the chromosome's unprotected end. This so-called “replicative senescence” or “telomere-induced senescence” triggers an enduring DNA damage response [34]. The DNA damage sensor protein, ATM, binds to these uncapped telomeres. This results in the stabilization of the tumor suppressor protein p53 and the increased activity of the p53-regulated gene p21^Cip1^. Consequently, p21^Cip1^ inhibits the activity of CDK2, crucial for preventing the deactivation of the Rb protein, blocking the cell’s progression into the S phase of its cell cycle, ultimately hindering DNA replication [35]. Interestingly, recent studies on bone marrow progenitor cells have highlighted that reoxygenation stress also activates the ATM-p53-p21^Cip1^ pathway, leading to a marked increase in cellular senescence. For example, Zhang et al. showed that reoxygenated bone marrow cells demonstrated a significant increase in ATM and p53 activation, with ~ 20% of stainings being positive for ATM-Ser1981 and p53-Ser15. Similarly, 22% of these cells expressed p21, revealing reoxygenation stress as another driver of DNA damage and cellular senescence [36]. Additionally, UV light, gamma irradiation, chemotherapy drugs (e.g., cyclophosphamide), and overactive oncogenic Ras proteins also cause DNA damage, leading to activation of the ATM-p53-p21^Cip1^ pathway [37]. Conversely, the p53-p21^Cip1^ pathway can be induced not only by responses to DNA damage but also through the expression of the p53 stabilizing protein p19Arf (known as p14 in humans), loss of the tumor suppressor protein PTEN, overexpression of the S phase transcription factor E2F3, or the upregulation of oncogenic Ras in human breast cells [38]. Further, inflammatory pathways have been identified as senescence drivers. Pro-inflammatory cytokines like IL-6 and TNF-α activate NF-κB signaling, promoting the transcription of SASP genes [39]. This phenotype is defined by different pro-inflammatory and tissue-destructive molecules, including a broad spectrum of chemokines, cytokines like IL-1α, IL-1β, and IL-8, the canonical inflammatory factor IL-6, and growth factors such as IGF2. SASP factors, especially IL-1β, IL-8, and MCP-1, create an autocrine loop reinforcing cellular senescence [40]. Moreover, hypoxia has been demonstrated to mediate senescence via the IL-6 axis in murine renal tissue hypoxia models. It is noteworthy that transplant surgery itself may also drive senescence through ischemia–reperfusion injury and the mechanosurgical trauma, among other pathways [41]. In experimental liver transplant models, for example, cellular senescence has been shown to be induced during organ retrieval and further exacerbated during static cold storage [2]. Other research has shown that cooling of cultured cells alone leads to increased production of reactive oxygen species (ROS), which cause double-strand breaks in DNA, subsequently inducing cellular senescence. These findings could be resembled when applying a standard clinical kidney transplant procedure to porcine kidneys [3]. In a murine cardiac IRI model, increased ROS levels mediated senescence of both cardiomyocytes and interstitial cell populations, contributing to impaired cardiac function and adverse tissue remodeling [4].

#### The effects of modulating senescence in solid organ transplantation

##### Reducing and dampening rejection reactions

Donor age has long been described as a significant risk factor for adverse outcomes after organ transplantation including delayed graft function and rejection [42, 43]. Mechanistically, this has been linked to increased immunogenicity of older organs. On a cellular level, high numbers of senescent cells have been found in old donor organs [13]. It has been shown that senescent cells are highly effective in triggering dendritic cells (DC) and antigen-specific CD8^+^ T cells [44]. In addition, impaired monocyte clearance of damaged necrotic cells has been observed with aging and may fuel inflammatory responses [45]. Ischemia–reperfusion injury (IRI), an event inevitably linked to transplantation, is also more pronounced in old organs. IRI has been shown to promote sterile inflammation by upregulating pro-inflammatory cytokines and inducing the formation of ROS that cause mitochondrial dysfunction [13]. A major cytokine released by senescent cells upon IRI is cell-free mitochondrial DNA (cf-mtDNA), which further promotes age-specific inflammatory responses. Congruent findings come from clinical studies of kidney transplant recipients, which showed that increased levels of mt-DNA had been linked to higher rates of acute rejection and delayed graft function [46]. Administering senolytics to old donor animals led to an attenuated cf-mtDNA increase after transplantation, subsequently dampening immune responses and promoting allograft survival in experimental models [18].

##### Leveraging synergistic effects with standard immunosuppressants

In addition to beneficial effects by directly targeting senescent cells and their products, synergistic effects of senolytics and standard immunosuppressive agents have been described. Calcineurin inhibitors such as tacrolimus and CsA are widely used immunosuppressants in SOT. Mechanistically, these drugs inhibit calcineurin phosphatase activity by binding to calcineurin, thereby preventing activation of nuclear factor of activated T cells (NFAT). This transcription factor, in turn, initiates IL-2 gene transcription, which induces T cell activation^48^. Interestingly, panabinostat, a senolytic drug and histone deacetylase inhibitor used in multiple myeloma therapy, has also been shown to target calcineurin. When tested in vivo, cotreatment with panabinostat and tacrolimus resulted in enhanced anti-tumor effects, thus suggesting synergistic effects when used for immunosuppression [47]. Furthermore, the tyrosine kinase inhibitor dasatinib has been observed to interfere with T cell receptor signaling by targeting the Src family. Moreover, additive effects with glucocorticoids such as dexamethasone on T cell suppression have been observed [48–50]. In addition, flavonoids such as quercetin and fisetin have been shown to inhibit the mTOR pathway, thus implying synergistic effects with mTOR inhibitors like rapamycin on T cell suppression [51]. Rapamycin is a senomorphic itself and has been shown to inhibit cellular senescence in vitro, improve lifespan in vivo, and suppress the SASP [52]. Moreover, Song et al. demonstrated direct suppressive effects of fisetin on T cell differentiation and proliferation in a dose-dependent manner [53]. Translating these findings into the clinic, co-administering immunosuppressive drugs and senolytics could allow for reduced dosages of immunosuppressants, thus ameliorating side effects such as increased susceptibility to infections and malignancies [54, 55].

### Preserving long-term graft function

Long-term outcomes of older organs have been shown to be compromised with reduced graft survival and increased rates of chronic allograft dysfunction across all types of transplants [56]. In kidney transplantation, for example, Oppenheimer et al. showed a linear increase of long-term graft failure and patient death with increasing donor age [57]. Histologically, markers of cellular senescence such as p16^INK4a^ have been associated with interstitial fibrosis and tubular atrophy, which contributed to late graft loss. Conversely, reducing the number of senescent cells by knocking out INK4a attenuated these changes and has been associated with conservation of nephron mass and prolonged allograft survival. In addition, IRI was less pronounced in INK4a deficient mice, supporting the important role of senescent cells in mediating IRI-induced damage [58, 59]. Supporting these findings, kidney biopsies from patients with tubular atrophy and interstitial fibrosis showed increased expression of p16^INK4a^ [60]. Moreover, investigating the long-term histology of transplanted livers, Rifai et al. described that donor age has been associated with higher levels of ductopenia and fibrosis in biopsies from patients ten years after transplantation. In contrast, donor age < 36 was a predictor for normal graft histology [61]. Taken together, these findings highlight that age and cellular senescence may not only pose a risk factor for short-term transplant outcomes but may also profoundly impair long-term graft function and survival rates. Eliminating senescent cells prior to transplantation by administering senolytics may thus improve long-term transplant outcomes.

### The senescence cascade in VCA tissues—identifying potential cellular levers and key molecules

#### Skin tissue

Skin tissue represents an integral part of VCA allografts, including a heterogenous set of alloreactive and immunotolerance-inducing cells [12]. Similar to the mucosa, skin tissue carries a high alloreactive potential [62–64]. Different senescence drivers can lead to distinct changes in skin composition and architecture. In the context of skin tissue senescence, particularly in VCA, several pathways interact to influence graft viability and function.

One such pathway is the JAK/STAT signaling pathway, which is closely linked to inflammatory responses in senescent skin cells. Activation of JAK/STAT by pro-inflammatory cytokines like IL-6 contributes to chronic inflammation in a senescent skin tissue. In particular, STAT3 activation amplifies the secretion of SASP components, reinforcing the pro-inflammatory state that characterizes senescent cells. This persistent inflammation not only compromises wound healing but also increases the antigenic profile of the skin graft, further heightening the risk of rejection [65]. In VCA settings, the JAK/STAT pathway plays a dual role—while it facilitates immune surveillance and host defense, its prolonged activation in senescent skin cells undermines the integrity and regenerative capacity of the graft, making it a critical target for modulating inflammation post-transplantation [66, 67].

Another key pathway involved in skin senescence is the mTOR signaling pathway, which regulates cell growth and metabolism. In senescent skin cells, mTOR is upregulated, contributing to the metabolic dysfunction often observed in aging tissues. mTOR activation promotes the production of SASP components, further driving inflammation and tissue degradation [68]. Additionally, mTOR signaling has been linked to delayed autophagy in senescent skin cells, impairing the clearance of damaged proteins and organelles. This accumulation of cellular debris accelerates the aging process, weakening the structural integrity of the skin [69]. The inhibition of mTOR has been explored as a potential therapeutic strategy to reduce senescence and enhance graft survival, particularly through the use of mTOR inhibitors like rapamycin. For instance, in murine models, combining low-dose IL-2 with rapamycin significantly prolonged skin allograft survival. This combination therapy extended graft survival from a median of 10 days in untreated controls to approximately 30 days in treated mice, effectively tripling the graft lifespan [70].

The p53/p21 pathway, known for its role in regulating the cell cycle and inducing senescence, also plays a critical role in skin tissue within VCA grafts. In response to DNA damage or oxidative stress, p53 is activated and promotes the expression of p21, a cyclin-dependent kinase inhibitor that halts the cell cycle and induces senescence [71]. In skin grafts, this pathway is particularly important for maintaining the balance between cellular proliferation and senescence. However, chronic activation of the p53/p21 axis in senescent skin cells can lead to a reduction in keratinocyte proliferation, weakening the skin barrier and impairing the graft’s ability to repair itself post-injury. This contributes to the thinning of the skin and makes the tissue more susceptible to mechanical stress, infections, and immune-mediated damage. In VCA patients, excessive activation of p53/p21 may also exacerbate graft fragility, leading to premature failure of the transplanted skin [72].

#### Mucosal tissue

Mucosal tissue plays a pivotal role in VCAs, especially in facial VCAs (fVCAs), where it constitutes a significant immunological interface and is hypothesized to influence both acute and chronic alloreactive rejection episodes. Recent studies have underscored the high alloreactive potential of mucosal tissue in VCA, demonstrating that the mucosal immune landscape can actively drive graft rejection processes in recipients [62–64, 73]. This dynamic may further be exacerbated by the SASP, which is a hallmark of senescent mucosal cells. Senescent mucosal cells secrete a plethora of pro-inflammatory cytokines such as interleukin-1β (IL-1β) and interleukin-6 (IL-6) [74], chemokines like CXCL1 and CXCL8 [40], and matrix-degrading enzymes like MMP-1 and MMP-3, creating a chronically inflamed microenvironment [75]. This inflammatory milieu is closely linked to the diminished regenerative capacity of senescent epithelial cells, contributing to tissue dysfunction and increasing susceptibility to rejection [29, 76].

A central mediator in mucosal senescence is interleukin-8 (IL-8)**,** a chemokine known for its dual role in promoting both inflammation and regeneration in epithelial tissues. In senescent mucosal cells, IL-8 interacts with the CXCR1 and CXCR2 receptors, initiating a signaling cascade through the NF-κB pathway. This pathway plays a pivotal role in driving the SASP and is responsible for recruiting immune cells—particularly macrophages and neutrophils—to sites of senescent mucosal tissue [74, 77]. The influx of these immune cells exacerbates local inflammation and promotes tissue damage, creating a feedback loop that may intensify graft rejection risk in VCA recipients. In the context of mucosal grafts, this IL-8-mediated immune cell recruitment has been implicated in acute rejection episodes, where the robust inflammatory response initiated by senescent cells accelerates tissue damage and graft deterioration [78]. However, IL-8 is not purely destructive in mucosal tissues. It has been shown to promote epithelial cell proliferation and contribute to wound healing by interacting with CXCR1/2 receptors on mucosal progenitor cells. This dual role highlights the complexity of senescence in mucosal tissues, where IL-8 can paradoxically drive both inflammatory damage and regenerative processes [79]. In senescent tissues, the balance between these opposing forces can cause persistent inflammation, impairing the tissue’s ability to regenerate effectively and leading to a decline in mucosal barrier function post-transplant [80].

TGF-β is another critical factor in mucosal senescence, particularly through its dual role in promoting inflammation and fibrosis while also facilitating tissue repair. In senescent mucosal tissues, TGF-β is activated via Smad-dependent and non-Smad pathways, both of which drive fibrotic responses and chronic inflammation [81]. While TGF-β can enhance epithelial cell proliferation and differentiation under normal circumstances, its chronic activation in senescent cells leads to excessive ECM deposition and fibrosis, which are hallmark features of long-term graft dysfunction in VCA recipients [81, 82]. Moreover, chronic TGF-β signaling disrupts normal mucosal architecture, thickening the ECM and reducing epithelial cell turnover, which is essential for maintaining tissue homeostasis [83]. Persistent activation of TGF-β also triggers the secretion of additional SASP factors, further perpetuating the senescence-associated inflammatory state in the mucosa [84, 85]. Studies have shown that Smad2/3 phosphorylation, a key step in TGF-β signaling, is significantly elevated in senescent mucosal cells, correlating with increased fibrosis and reduced tissue function in transplanted mucosal tissues. This chronic activation of TGF-β also leads to a loss of epithelial stem cell function, thereby limiting the mucosa’s ability to regenerate and repair post-transplant, ultimately increasing the risk of long-term graft failure [86–88].

Another key pathway that is altered in senescent mucosal tissues is the Notch signaling pathway. In the context of senescent mucosal tissues, disruptions in Notch signaling lead to impaired differentiation and proliferation of mucosal progenitor cells, which are essential for maintaining epithelial barrier integrity and promoting tissue repair [89, 90]. Studies have demonstrated that senescent mucosal progenitor cells exhibit downregulated expression of Notch receptors and ligands, such as Notch1 and Jagged1, leading to reduced regenerative capacity and delayed wound healing in VCA mucosal grafts. This downregulation compromises the activation of target genes like Hes1, which is responsible for progenitor cell maintenance and differentiation towards goblet cells, further inhibiting proper mucosal healing [91–93].

#### Muscle tissue

Muscle tissue is integral for restoring functionality in patients undergoing VCA surgery, reanimating facial expression and extremity movement [94–96]. One critical pathway implicated in muscle senescence is the inflammatory cascade driven by IL-6, TNF-α, and TGF-β, which accelerates muscle protein breakdown and fibrosis. These inflammatory cytokines act through the NF-κB and MAPK pathways, contributing to chronic inflammation, myogenic differentiation impairment, and tissue remodeling [97]. Specifically, MMPs induced by these pathways degrade muscle extracellular matrix, promoting fibrosis, and thus reducing muscle elasticity and contractile ability. This inflammation-fueled cascade has been shown to persist in senescent muscle, leading to poor graft outcomes due to impaired muscle function [98, 99].

Moreover, the cyclin-dependent kinase inhibitor p21 plays a crucial role in mediating muscle senescence. Studies have shown an age-related increase in p21 expression in skeletal muscle [5, 6]. Utilizing an overexpressing p21 mouse model, various characteristics of senescence, such as DNA and mitochondrial damage as well as upregulated SASP, could be resembled. Histopathological alterations included atrophy and fibrosis, which manifested in a decreased physical capacity in functional tests [7]. Accordingly, experimental suppression of p21 resulted in increased proliferative capacity of myocytes, further underlining its important role in muscle senescence and proliferation [8].

Another pathway interwoven with muscle tissue senescence is satellite cell dysfunction. Satellite cells, identified by the expression of surface markers such as paired box protein 7 (Pax7), CD56 (neural cell adhesion molecule, NCAM), and myogenic factor 5 (Myf5), are muscle-specific stem cells essential for muscle repair and regeneration. A decline in both the number and functionality of satellite cells is a hallmark of advancing senescence [100, 101]. This reduction is driven by intrinsic factors like telomere shortening and extrinsic influences, including chronic inflammation and oxidative stress. The natural senescence of satellite cells hampers muscle regeneration after transplantation, ultimately impacting long-term graft functionality [102].

Finally, mitochondrial dysfunction is a hallmark of muscle senescence that severely affects satellite cell efficacy. The AMPK/SIRT1 pathway regulates mitochondrial health, and its decline in aging cells leads to decreased mitochondrial biogenesis and increased ROS (reactive oxygen species) production. This results in disrupted energy production, which impairs the regenerative capacity of satellite cells [103, 104]. Moreover, mitochondrial dysfunction triggers apoptosis through the cytochrome c-caspase-9-caspase-3 pathway, further exacerbating muscle tissue loss. This apoptotic process reduces the muscle’s contractile function, affecting the overall functionality of the VCA graft [105, 106]. Additionally, fibrosis induced by muscle apoptosis leads to the excessive deposition of ECM components by activated fibroblasts. This fibrotic response alters the mechanical properties of the muscle, limiting its functional capacity and volume, reducing graft endurance over time [107, 108].

#### Bone tissue

Bone tissue serves as the primary load-bearing compound of VCA grafts and is critical for the graft’s overall stability [109]. Cellular senescence within bone tissue has been linked with impaired bone quality and mineralization, potentially compromising the mechanical stability and structural integration of the graft [110]. One of the key pathways that promotes senescence in bone tissue is the RANKL (Receptor Activator of Nuclear Factor Kappa-Β Ligand) axis, which regulates osteoclastogenesis and bone resorption. Senescent bone cells, especially osteoclasts and osteoblasts, exhibit elevated levels of pro-inflammatory cytokines such as IL-1, IL-6, and TNF-α, which amplify the RANKL signaling cascade. These cytokines enhance osteoclast differentiation and activity, leading to excessive bone resorption while simultaneously inhibiting osteoblastic bone formation [9, 10]. This imbalance in bone turnover results in reduced bone density, mimicking osteoporotic conditions, which in turn predisposes VCA graft recipients to fractures and mechanical instability [111, 112]. Studies have shown that elevated RANKL/OPG ratios (osteoprotegerin being the decoy receptor for RANKL) are directly associated with accelerated bone loss in senscent cells  [111–115].

Another crucial factor in bone tissue senescence is the p53/p21 and p16^INK4a^ pathways, which are well-established regulators of cellular senescence. These pathways are upregulated in senescent osteoblasts and MSCs, leading to a significant decrease in the osteogenic potential of these cells. Senescent osteoblasts are characterized by a reduction in key osteogenic markers, such as Runx2 and collagen type I, essential for bone matrix synthesis and mineralization [26, 116]. Moreover, these cells exhibit increased activity of SA-β-gal. This osteoblast dysfunction leads to impaired bone remodeling and the potential for non-union or pseudarthrosis in VCA grafts. GDF15 (Growth Differentiation Factor 15) has been shown to exacerbate this process by inhibiting osteoblast differentiation, further contributing to reduced bone formation [110, 117].

A third key pathway contributing to senescence in bone tissue involves MSCs and their shift towards adipogenic differentiation at the expense of osteoblastogenesis. In senescent MSCs, Peroxisome Proliferator-Activated Receptor Gamma (PPARγ) signaling is upregulated, promoting adipocyte formation and leading to bone marrow fat accumulation [118, 119]. This shift not only depletes the pool of osteoblast progenitors but also results in the infiltration of adipocytes into the bone marrow space, altering bone architecture and reducing bone density [120]. The decline in osteoblastic differentiation capacity and the increase in bone marrow adiposity are well-documented contributors to the compromised structural integrity of VCA grafts [121]. These changes can impair the integration of bone with surrounding tissues and lead to a weakened skeletal framework in the graft.

#### Nerve structures

Nerve structures are pivotal compounds to restore sensory and motor function in VCA grafts [122]. In senescent nerve tissue, several critical pathways become dysregulated, impairing the ability of nerves to regenerate and perform their essential roles in sensory and motor recovery. Senescence-driven changes are particularly prominent in pathways related to inflammation, regeneration, and neurotrophic support, which collectively degrade nerve function and compromise graft outcomes [123, 124].

One primary pathway implicated in nerve senescence is the p38 MAPK pathway, which plays a dual role in both initiating acute inflammatory responses and perpetuating chronic inflammation in senescent cells. Under normal physiological conditions, p38 MAPK activation is critical for Wallerian degeneration, a process essential for clearing axonal debris after nerve injury [125]. However, in senescent nerves, persistent activation of p38 MAPK has been shown to exacerbate the release of SASP factors, such as IL-6 and TNF-α [126]. Chronic activation of p38 MAPK suppresses the regenerative capacity of nerves by enhancing the pro-inflammatory milieu, thereby prolonging tissue injury and inhibiting Schwann cell-mediated axonal repair. This prolonged activation has also been linked to a decline in Schwann cell plasticity, further compromising the nerve’s ability to remyelinate damaged axons and restore proper function in VCA patients [125, 127].

A second critical pathway involved in nerve senescence is the NF-κB pathway, which is also upregulated in senescent nerve cells. In the context of nerve injury and aging, NF-κB becomes persistently activated, driving the expression of pro-inflammatory genes, including IL-1, COX-2, and cell adhesion molecules like ICAM-1 and VCAM-1 [128]. This chronic activation exacerbates inflammation and impairs nerve regeneration by promoting leukocyte infiltration, which not only delays healing but also increases the risk of scar formation and fibrosis in nerve tissues [129]. Studies show that NF-κB activity in senescent Schwann cells disrupts their ability to form supportive niches for axonal growth, further hindering peripheral nerve regeneration. Additionally, NF-κB is a key regulator of Wallerian degeneration, and its dysregulation can impair the clearance of axonal debris, preventing efficient nerve repair in VCA grafts [130, 131].

The JAK/STAT (Janus Kinase/Signal Transducers and Activators of Transcription) pathway is another signaling cascade highly relevant to nerve senescence. This pathway regulates cytokine signaling and cellular responses to inflammation, injury, and stress. In senescent nerve tissues, JAK/STAT signaling is often dysregulated, leading to excessive production of inflammatory cytokines and growth factors, which further impede nerve regeneration [132]. The role of SOCS3 (Suppressor of Cytokine Signaling 3) in inhibiting regenerative signals from neurotrophic factors has been shown to hinder nerve healing. For instance, in senescent neurons, the overexpression of SOCS3 blocks the effects of BDNF (Brain-Derived Neurotrophic Factor) and other neurotrophins, impeding axonal outgrowth and repair. This pathway’s dysregulation in senescent nerve tissue diminishes the regenerative potential of peripheral nerves, contributing to poor sensory and motor recovery following VCA procedures [133].

Beyond these signaling pathways, senescence-related declines in neurotrophin production represent a critical impediment to nerve regeneration. Neurotrophins, including NGF (Nerve Growth Factor), BDNF, and NT-3 (Neurotrophin-3), are vital for the survival, growth, and differentiation of neurons, particularly in the context of nerve injury and repair. In senescent nerves, there is a significant reduction in the production of these neurotrophins [134]. For instance, NGF, which is essential for the survival and maintenance of sensory and sympathetic neurons, binds to TrkA receptors to promote neuronal survival and axonal growth [135]. However, in senescent tissues, the downregulation of NGF and its signaling impairs axonal regeneration, leaving damaged nerves less capable of reinnervating graft tissues and restoring function [136].

#### Arterial and venous vasculature

Vasculature is critical in VCAs because it ensures the necessary blood, oxygen, and nutrient exchange in transplanted tissues [137]. The arterial vasculature, serving as the primary conduit for oxygenated blood and nutrients, is fundamentally regulated by pro-angiogenic factors like vascular endothelial growth factor (VEGF) and fibroblast growth factor (FGF). These growth factors are essential for the survival, proliferation, and migration of endothelial cells, which form the inner lining of the arterial wall [138]. Their actions are mediated through the binding to specific receptor tyrosine kinases, such as VEGFR and FGFR, leading to the activation of downstream signaling cascades like the PI3K/Akt pathway. This signaling pathway is critical for mediating cellular processes central to angiogenesis and vascular repair [139]. In senescent endothelial cells, studies have shown a marked decrease in the expression and activity of these growth factors and their receptors, which disrupts key signaling cascades, such as the PI3K/Akt pathway. The downstream effects of this disruption include impaired angiogenesis and vascular repair mechanisms, both of which are vital for the integration of transplanted tissues into the recipient’s circulatory system [140–142].

Senescent endothelial cells also exhibit decreased capillary density by up to 30–50% and reduced angiogenic potential, leading to diminished blood flow and oxygen delivery to graft tissues [143, 144]. This decline in physiological capacity is particularly problematic in the context of VCAs, where the rapid revascularization of the transplanted tissue is necessary for graft survival. Experimental models of senescent endothelial cells demonstrate that the reduction in VEGF and FGF signaling leads to poor re-endothelialization following injury, further exacerbating the challenges of maintaining graft perfusion [143]. In addition, hypoxia, a common consequence of vascular insufficiency, is a potent modulator of cellular senescence. Hypoxia-induced activation of Hypoxia-Inducible Factor (HIF) and the subsequent increase in ROS levels can accelerate senescence pathways in endothelial cells, creating a vicious cycle of vascular decline and tissue hypoxia [145].

Additionally, the expression of endothelial nitric oxide synthase (eNOS), which is responsible for the production of nitric oxide (NO), is diminished in senescent endothelial cells. This decrease in NO production leads to vasoconstriction, impaired blood flow, and increased vascular resistance in the transplanted tissues. The resultant endothelial dysfunction promotes thrombosis, increases vascular permeability, and heightens the risk of graft failure [146].

Chemokines such as CCL2 and IL-8 further fuel this inflammatory cascade by recruiting immune cells, including CD8^+^ T cells, natural killer (NK) cells, and macrophages, to the site of vascular injury. These immune cells amplify the local inflammatory response, creating a feedback loop that perpetuates vascular inflammation and senescence. IL-8 has been particularly implicated in promoting the migration and activation of neutrophils, contributing to vascular damage and hindering tissue repair [147]. The persistent upregulation of these inflammatory mediators not only compromises vascular integrity but also heightens the risk of graft rejection and vascular complications in VCA recipients [148].

**Fig. 1:**
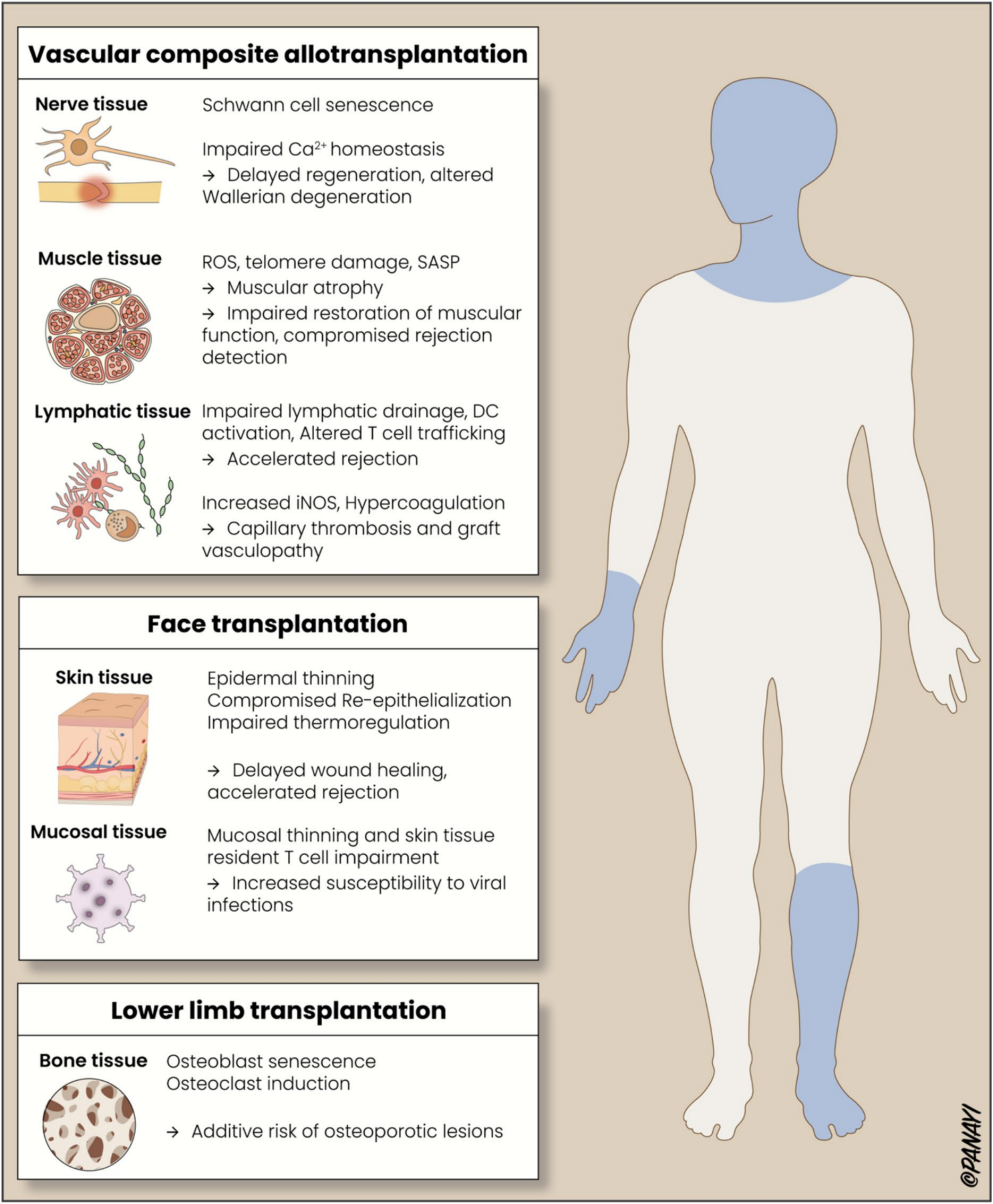
The impact of senescence on VCA tissues. Senescence has been shown to induce different macro- and microanatomical shifts in VCA tissues. For example, senescence has been implicated with mucosal and epidermal thinning, as well as impaired muscle and nerve regeneration [149, 150]. In the lymphatic tissue, senescence has been associated with poor lymphatic drainage [151]

An overview of pathways involved in senescence across tissues relevant to VCA, as well as the impact of senescence on VCA tissues, is depicted in Table 1 and Fig. 1, respectively.

**Table 1:** Summary of pathways involved in senescence across specific tissues relevant to VCA. Summary of the primary pathways involved in senescence across various tissue types commonly found in VCA, including skin, mucosal, muscle, bone, nerve, and vascular tissues

Tissue	Most important pathways of senescence	Effects of the pathways
Skin	JAK/STAT mTOR p53/p21	Modulates inflammation and regeneration; chronic activation impairs repair
Mucosal	IL-8/NF-κB TGF-β**/**Smad Notch	Drives inflammation and tissue repair; chronic activation promotes fibrosis
Muscle	NF-κB/MAPK Satellite cell senescence Mitochondrial dysfunction	Affects muscle regeneration and repair; chronic activation leads to tissue degeneration
Bone	RANKL/OPG p53/p21 p38 MAPK/PPARγ	Regulates osteoclastogenesis and bone turnover; imbalance leads to bone weakening
Nerve	p38 MAPK NF-κB JAK/STAT	Involved in nerve inflammation and repair; chronic activation inhibits nerve regeneration
Vasculature	PI3K/Akt NF-κB Shear stress/mechanotransduction	Regulates angiogenesis and function; dysfunction affects blood flow and tissue integration

## Discussion

Over the past two decades, VCA surgery has emerged as a novel surgical approach to target extensive tissue defects. There is a body of evidence underlining the positive functional and reconstructive outcomes after VCA surgery [7] Beyond outcome research, VCA providers have investigated various aspects of this biotechnology ranging from molecular pathways over laboratory rejection markers to ethical challenges [1, 3, 12].[11–13] This review highlights senescence as a promising field for future VCA research. However, senescence-induced tissue changes remain to be investigated in VCA rodent models and human tissues.

Previous SOT research has demonstrated the untapped therapeutic potential of senescence to expand the transplant donor pool, reduce and dampen rejection reactions, leveraging synergistic effects with immunosuppressive drug regimes, and preserving long-term graft function [152]. Senolytics have been proposed to modulate senescence and selectively eliminate senescent cells. Mechanistically, senescence-associated antiapoptotic pathways (SCAPs) protect senescent cells from undergoing apoptosis. Senolytics inhibit SCAPs to reverse apoptosis resistance and enable clearance by immune cells. Since senescent cells exhibit multiple SCAPs, combination treatment with different senolytics is usually applied [73]. Various animal studies have shown that senolytics can reverse aging phenotypes, including organ-specific functions such as kidney function [76]. Moreover, senolytics increased lifespan and healthspan in aged animals [73]. Of relevance for transplantation, treating old donor animals with senolytic drugs diminished ischemia–reperfusion injury and led to a significant improvement of allograft survival. Transplanting senescent cells directly into the abdominal cavity of young animals or correspondingly transplanting older donor organs to young recipients induced age-related phenotypes with augmented SASP factors and compromised physical as well as cognitive capacities, whereas eliminating these cells by senolytics reversed the changes [18, 19, 24]. Different types of senolytics have been investigated in experimental organ transplant models. Administering Dasatinib plus Quercetin (D + Q) to donor animals prior to cardiac transplantation has led to a decrease in senescent cell burden as well as improved physical fitness of recipient mice after transplantation [14]. Moreover, administering the senolytic ABT-737 in liver regeneration models leads to decreased p21 expression, thus leading to better functional outcomes by promoting liver regeneration. Adding the same drug to the perfusate of discarded human liver grafts ameliorated biliary damage and improved regenerative capacity of hepatocytes [15]. Furthermore, rapamycin, a senomorphic agent, suppressed allograft senescence and SASP levels in a rat kidney transplant model. By reducing interstitial fibrosis and tubular atrophy, this may protect grafts from post-transplant functional decline^17^.

**Fig. 2:**
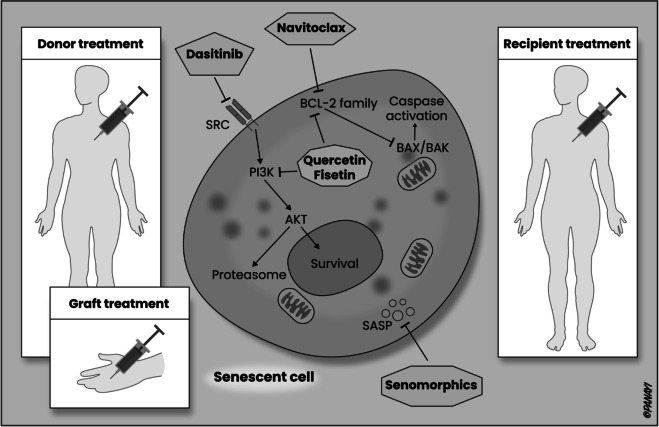
Pharmacological senolytic strategies. Different receptors, proteins, and molecules have been discussed as potential targets for senolytic strategies. For instance, senolytics can inhibit tyrosine kinases, growth factor receptors, or members of the BCL-2 family [155–157]. Further, senomorphics such as telomerase activators cycloastragenol (TA-65) and resveratrol or mTOR inhibitor rapamycin have been demonstrated to modulate the SASP by interfering with common senescence triggers (e.g., radiation, toxins) [23, 24] [158]

**Fig. 3:**
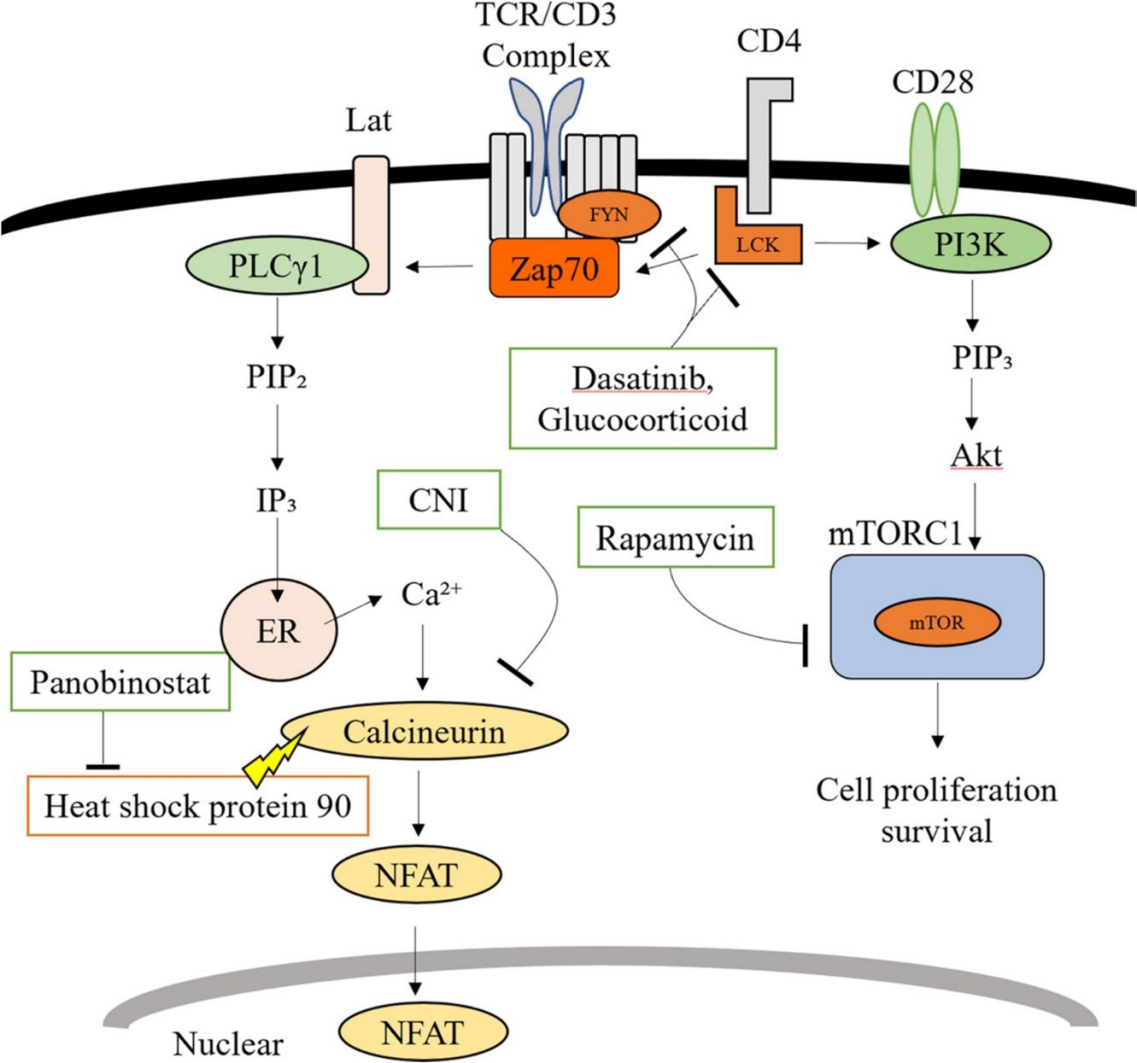
Synergistic effects of senolytics and senomorphics with standard immunosuppressants. Panobinostat, a senolytic agent used in multiple myeloma therapy, and calcineurin inhibitors such as tacrolimus and CsA synergistically prevent NFAT activation, suggesting enhanced immunosuppressive effects when combined in vivo [47, 159]. Dasatinib and glucocorticoids both interfere with T cell receptor signaling, and additive immunosuppressive effects on T cells have been demonstrated [48–50]. Rapamycin, a senomorphic drug, both prevents cellular senescence and mediates T cell suppression [51, 52]

Experimental studies, including heterochronic parabiosis models, have shown that aging satellite cells can be rejuvenated by systemic factors present in young blood, underscoring the potential of external interventions to reverse senescence-related impairments in satellite cells. For example, SSK1, a targeted senolytic, selectively diminished and reduced SA-βgal + cell count by 3.8-fold in mouse lung injury and lowered senescence gene expression measured via RT-PCR. Additionally, SSK1 outperformed senolytics like ABT263 and dasatinib plus quercetin, boosting grip strength and treadmill capacity [153]. In addition to experimental studies, data from a first in-human clinical trial investigating the potential of senolytics in patients with idiopathic pulmonary fibrosis (IPF) have shown that senolytics could attenuate physical dysfunction [154]. Currently, clinical trials are underway to investigate the safety and effectiveness of senolytics in a variety of other diseases such as chronic kidney disease (CKD), lung fibrosis, Alzheimer’s disease, and COVID-19 [73] (Figs. 2 and 3).

Translating these findings into VCA may help overcome persisting challenges in VCA. Expanding the donor pool could potentially reduce patients’ wait times for VCA transplants, which, in cases like facial transplantation, can vary from 1 day to 17 months [160]. Longer waiting times have been implicated with poorer postoperative outcomes in SOT. For example, Gill et al. analyzed 63,783 kidney transplant candidates and found that increased waiting times from one to three years significantly decreased the overall quality of life benefit of transplantation [161]. Similarly, Beumer et al. reviewed 45,694 liver transplant patients and reported that the median time to postoperative liver failure reduced by 3.41 years for patients waiting twelve months versus those waiting only two months [162]. While the link between waiting times and VCA outcomes remains to be investigated, research from SOT clearly points towards a scientific foundation that shorter wait times may translate into improved patient outcomes. Moreover, a broader donor pool may facilitate skin color matching in Black VCA patients, ensuring racial and ethnic inclusivity in this patient cohort [2, 160]. Modulating senescence in SOT has further been demonstrated to prevent rejection episodes and promote transplant longevity. Nearly 85% of VCA patients experience at least one episode of acute reaction, with more than 50% of cases reporting multiple rejection episodes [10]. Acute and chronic rejections have been associated with impaired VCA graft function, including necrosis and GV [5, 9]. While the Yale and Paris groups demonstrated the feasibility and safety of VCA retransplant, preventing rejection episodes represents a cornerstone to optimize VCA outcomes [163, 164]. Further, the findings derived from SOT suggest that the combination of senolytics with immunosuppressants may allow a reduction in dosages of immunosuppressive drugs. This approach may reduce the rate of immunosuppressant-induced side effects (e.g., malignancies, kidney/liver failure, and metabolic disorders) [165].

Senescence remains a cellular process regulating human aging with physiological implications for maintaining tissue homeostasis including stress response modulation, metabolic regulation, tissue regeneration, and the prevention of tumor formation and fibrosis [166]. Rather than eliminating senescent cells per se, targeting a state of “senescent homoestasis” which balances physiological effects on organ function while limiting the detrimental impact on ischemia reperfusion injury and allo-immune responses may constitute the ultimate therapeutic goal. Therefore, future studies are required to delineate the impact of clearing senescent cells in transplantation not only on short-term immunological outcomes but also long-term performance respecting organ function and quality despite the detrimental effects of immunosuppressants. Recent studies investigating the impact of senolytics in transplantation have achieved significant reduction of senescent cells in donor organs; however, without a full depletion [18]. Identifying thresholds of pathological senescent cell burdens will be necessary to define therapy endpoints for senolytic treatment. Moreover, the transplant process itself has been shown to induce senescence in recipient organs as well as the recipient immune system [167, 168]. Therapeutic approaches may thus also involve the inhibition of senescence potentiation following transplantation.

In the context of VCAs, senescence pathways reveal complex, tissue-specific roles that oscillate between repair and pathology [169]. The NF-κB pathway is a central regulator of chronic inflammation across skin, mucosal, nerve, and vascular tissues [170]. While essential for early immune activation through cytokines like IL-6, TNF-α, and IL-1β, its chronic activity fosters a pro-inflammatory milieu that impedes repair and drives fibrosis, particularly in vascular and nerve tissues through leukocyte adhesion and migration [171].

The p38 MAPK pathway similarly exerts context-dependent effects. It promotes Wallerian degeneration in acute nerve injury but, under chronic conditions, perpetuates inflammation and SASP secretion in muscle, nerve, and skin, exacerbating tissue damage [125]. TGF-β signaling underscores this duality: it supports cell proliferation and repair in mucosal and muscle tissues but induces fibrosis and chronic inflammation through Smad and non-Smad pathways when persistently activated [172, 173].

JAK/STAT signaling compounds the complexity in senescent nerve and vascular tissues, leading to cytokine overproduction and suppression of neurotrophic pathways via SOCS3, impairing NGF and BDNF-mediated nerve regeneration and myelination [174, 175]. Notch signaling, crucial for progenitor cell differentiation, is similarly downregulated in senescent tissues, limiting regenerative potential and delaying wound healing [176].

These pathways intersect and sometimes counteract each other. NF-κB and p38 MAPK reinforce chronic inflammation and fibrosis, while TGF-β and Notch can toggle between regenerative and fibrotic roles [177–179]. The suppression of neurotrophic pathways by JAK/STAT contrasts with the pro-regenerative nature of NGF and BDNF, illustrating how senescence-driven mechanisms simultaneously stifle repair while maintaining inflammatory and fibrotic states [180].

Sex has been shown to be another important factor impacting cellular senescence. For example, differences in telomerase activity, DNA-damage repair, and hormonal levels have been shown to be of relevance in the context of aging [17–21]. In solid organ transplantation, sex has been observed to impact transplant outcomes in an age-dependent fashion, suggesting potential relevance also in the field of VCA [22]. However, there is currently little evidence on the interplay of sex and cellular senescence in VCA, and further research is needed to investigate its effects.

This line of research highlights the body of evidence on senescence in SOT and aims to discuss the potential implications for the field of VCA. Modulating cellular senescence holds promising potential to mitigate immune rejection and improve outcomes in VCA. While senescence-targeting strategies, such as senolytics, have demonstrated efficacy in SOTs, their application, therapy sequence, timing, treatment duration, and combination in VCA remain an open field for exploration. In this context, the role of chronological and biological age in VCA surgery remains to be investigated, with previous research on SOT highlighting the relevance of differentiating between chronological and biological age [181]. Future research into senescence modulation could uncover strategies to reduce rejection episodes and extend graft longevity in VCA patients, paving the way for advancements that may significantly transform long-term management and outcomes in this complex transplant field. Our in vivo findings demonstrate that donor tissues with a high burden of senescent cells, such as those from aged organs, can trigger senescence in recipient cells following transplantation. This process results in increased systemic inflammation, elevated release of mitochondrial DNA, and a decline in physical and cognitive functions. In contrast, tissues from younger donors or those pretreated with senolytics, which have fewer senescent cells, elicit less systemic aging and better preserve the recipient’s functional capacities. These results highlight that the senescent cell load in donor organs directly impacts recipient outcomes [167]. Additionally, we found that cell-free mitochondrial DNA, released by senescent cells, accumulates with age and enhances immunogenicity in vivo. Clinically, elevated levels of cf-mt-DNA in older donors correlate with activation of human dendritic cells. In a murine heart transplant model, senolytic treatment of aged donor animals effectively reduced senescent cell burden and cf-mt-DNA release, dampened age-specific immune responses, and prolonged graft survival to levels comparable to those of young donor organs [18]. However, current research provides an isolated viewpoint zooming in on one specific tissue yet zooming out on the complex tissue interaction in VCA grafts. Therefore, senescence remains to be investigated in VCA rodent models and human tissues. Future studies may elucidate the cross-link of senescence and VCA to exploit the currently untapped therapy potential and advance these two research fields.

## Abbreviations

*ADMSC*: Adipose Tissue-Derived Stromal/Stem Cell
*ADSC*: Adipocyte-Derived Stem Cells
*AMPK*: AMP-Activated Protein Kinase
*ATM*: Ataxia Telangiectasia Mutated
*BDNF*: Brain-Derived Neurotrophic Factor
*BMAT*: Bone Marrow Adipose Tissue
*BMSCs*: Bone Marrow-Derived Stem Cells
*CCL*: Chemokine (C–C motif) Ligand
*CDK2*: Cyclin-Dependent Kinase 2
*CXCL*: C-X-C Motif Chemokine Ligand
*CXCR*: C-X-C Chemokine Receptor
*CON*: Control Non-Senescent
*CsA*: Cyclosporin A
*DC*: Dendritic Cell
*DNA*: Deoxyribonucleic Acid
*eNOS*: Endothelial Nitric Oxide Synthase
*ERK*: Extracellular Signal-Regulated Kinase
*FAP*: Fibroadipogenic Progenitor Cells
*FGF*: Fibroblast Growth Factor
*FGFR*: Fibroblast Growth Factor Receptor
*GV*: Graft Vasculopathy
*HDF*: Human Dermal Fibroblast
*HGF*: Hepatocyte Growth Factor
*HIF*: Hypoxia-Inducible Factor
*ICAM*: Intercellular Adhesion Molecule
*IGF2*: Insulin Growth Factor 2
*IL*: Interleukin
*IPF*: Idiopathic Pulmonary Fibrosis
*iNOS*: Inducible Nitric Oxide Synthase
*IRI*: Ischemia-Reperfusion Injury
*JAK*: Janus Kinase
*MAPK*: Mitogen-Activated Protein Kinase
*MBP*: Myelin Basic Protein
*MCP*: Monocyte Chemoattractant Protein
*MHC*: Major Histocompatibility Complex
*MMP*: Matrix Metalloproteinases
*mTOR*: Mechanistic Target of Rapamycin
*NFAT*: Nuclear Factor of Activated T-Cells
*NF-κB*: Nuclear Factor Kappa-Light-Chain-Enhancer of Activated B Cells
*NGF*: Nerve Growth Factor
*NK*: Natural Killer
*NO*: Nitric Oxide
*NT-3*: Neurotrophin-3
*OPG*: Osteoprotegerin
*P0*: Myelin Protein Zero
*p16INK4A*: Cyclin-Dependent Kinase Inhibitor 2A
*p19Arf*: P19 Alternative Reading Frame (known as p14ARF in humans)
*p21Cip1*: Cyclin-Dependent Kinase Inhibitor 1
*PMP22*: Peripheral Myelin Protein 22
*PPARγ*: Peroxisome Proliferator-Activated Receptor Gamma
*PRR*: Pattern Recognition Receptors
*PTEN*: Phosphatase and Tensin Homolog
*RANKL*: Receptor Activator of Nuclear Factor Kappa-Β Ligand
*Rb*: Retinoblastoma Protein
*ROS*: Reactive Oxygen Species
*SA-β-Gal*: Senescence-Associated Beta-Galactosidase
*SA-βgal*: Senescence-Associated Beta-Galactosidase
*SASP*: Senescence-Associated Secretory Phenotype
*SCAP*: Senescence-Associated Antiapoptotic Pathways
*SEN*: Senescent
*SIRT1*: Sirtuin 1
*SMCs*: Smooth Muscle Cells
*SOT*: Solid Organ Transplantation
*SOCS3*: Suppressor of Cytokine Signaling 3
*STAT*: Signal Transducer and Activator of Transcription
*TGF-β*: Transforming Growth Factor Beta
*TNF-α*: Tumor Necrosis Factor Alpha
*Treg*: Regulatory T Cell
*Tr1*: Type 1 Regulatory Cell
*UCP1*: Uncoupling Protein 1
*VCAM*: Vascular Cell Adhesion Molecule
*VCA*: Vascularized Composite Allotransplantation
*VEGF*: Vascular Endothelial Growth Factor
*VEGFR*: Vascular Endothelial Growth Factor Receptor
